# Using Structured Observation and Content Analysis to Explore the Presence of Older People in Public Fora in Developing Countries

**DOI:** 10.1155/2014/860612

**Published:** 2014-12-08

**Authors:** Geraldine Nosowska, Kevin McKee, Lena Dahlberg

**Affiliations:** ^1^Effective Practice Ltd., 8 Bridgetown, Totnes, Devon TQ95AB, UK; ^2^School of Education, Health and Social Studies, Dalarna University, 791 88 Falun, Sweden; ^3^Aging Research Centre, Karolinska Institutet and Stockholm University, Gävlegatan 16, 113 30 Stockholm, Sweden

## Abstract

There is a lack of research on the everyday lives of older people in developing countries. This exploratory study used structured observation and content analysis to examine the presence of older people in public fora and considered the methods' potential for understanding older people's social integration and inclusion. Structured observation occurred of public social spaces in six cities each located in a different developing country and in one city in the United Kingdom, together with content analysis of the presence of people in newspaper pictures and on television in the selected countries. Results indicated that across all fieldwork sites and data sources, there was a low presence of older people, with women considerably less present than men in developing countries. There was variation across fieldwork sites in older people's presence by place and time of day and in their accompanied status. The presence of older people in images drawn from newspapers was associated with the news/non-news nature of the source. The utility of the study's methodological approach is considered, as is the degree to which the presence of older people in public fora might relate to social integration and inclusion in different cultural contexts.

## 1. Introduction

By 2050, there will be approximately 2 billion people aged 60 years or more and the number of older people will outnumber the young. The most rapid changes will take place in developing countries, and projections indicate that by then four of five older people will live in developing countries [1]. This demographic change has implications not only for the delivery of health and social care and for age-related policy issues, such as pensions, but also for the everyday life of citizens both young and old. Our capacity to understand how demographic changes in developing countries will impact on older people's lives is limited by the relative lack of research on the everyday lives of older people in such countries [2]. In this paper, we describe an exploratory study that examines the public presence of older people, with consideration of differences between women and men. This is done via the structured observation of social spaces in selected cities in six developing countries and one city in a developed county and content analysis of examples of the countries' respective print and broadcast media, specifically images in newspapers and on television channels. The potential of such data for understanding the social integration and inclusion of older people is discussed.

When proposing a need for more social research on older people in developing countries, the methodological approach to be adopted requires particularly careful consideration. Applying conventional research methods might be problematic. Using survey methods and instruments created for use in developed countries raises issues of validity, if concepts and language are simply imported without thought for differing cultural contexts. Difficulties in accessing older people and the use of standardised but culturally biased procedures and formats for delivering questions and eliciting responses threaten reliability and representativeness (cf. [3]). Qualitative methods are suitable for developing understanding and theory and are arguably more sensitive than quantitative studies to cultural issues. However, studies using such methods have limitations as their (usually) small and unrepresentative samples prevent generalization of their findings to target populations, and the studies' findings are additionally often difficult to reproduce [4].

Observational methods and content analysis might circumvent some of the problems that arise in using conventional research methods in the context of developing countries. It can be argued that the actual presence or absence of older people in public fora, such as social and cultural spaces, is a good marker of their level of social integration (cf. [5]). The use of social spaces can influence societal attitudes to different groups and the degree to which they belong in society [6]. Also, it can direct us towards the way space works to include or exclude people [7]. Presence or absence can also be considered through visibility in different media such as newspapers, films, or television. Mass media are a force for shaping attitudes and images of older people in media shape perceptions and self-perceptions of ageing [8–11]. Underrepresentation in media could lead to a perception that groups of the population are of little significance to society (cf. [12]). Thus, how far older people are visible in such cultural spaces and the nature of their presentation are both reflections and drivers of social norms, becoming both measures of social integration and factors that affect social integration.

Studies that document the presence or absence of older people in public fora might therefore provide valuable data that will contribute to our understanding of the place of older people in their respective societies. However, there are a very limited number of such studies, particularly in developing countries. There is some information, primarily from developed countries, about places that are important to older people. For example, Londoners identified a religious building as an important place in their lives [13] and different ethnic groups identified varying benefits in using parks in the United States of America (US) [14], while older people in Hong Kong found that poor health, lack of time, and issues with other users reduced their use of parks, despite their health and social and psychological benefits ([15]; cf. [16]).

With regard to the presence and form of presentation of older people in broadcast and print media there is a considerable body of work available, but there is a knowledge gap around images of older people in non-Western cultures with cross-cultural research extremely limited [17]. In the available research from developed countries, there is a consensus that older people have been and continue to be less present than expected with regard to their prevalence in the population. For example, an extensive observational study of German television networks found that people over 60 were very underrepresented, particularly women and those of advanced old age [18]. Research on prime time US television echoes this [19–21]. Similarly, US [22] and German studies [23] have found an underrepresentation of older people in television and printed advertisements, respectively. Content analysis of print media in one area in the USA found that stories that could be illustrated by pictures of people of any age tended to feature younger people [24]. Older people were also underrepresented in the United Kingdom (UK) magazine advertisements, Canadian television advertisement, and Irish newspapers [25–27]. Although there are positive images on older people in aspirational advertising (for a discussion, see [27]), when present in media older people are frequently constructed as victims and being frail, vulnerable, and so forth [28], not the least when mentioned in the context of population ageing [9].

Considering developing or non-Western countries, a study on the presentation of older people on Taiwanese television [29] produced findings similar to those obtained in the USA, while studies looking at the presentation of older people in the media in India and Japan found that there was an underrepresentation of older people, particularly of older women, in magazine and television announcements, respectively [30, 31].

When studying older people's presence in public fora, it is important to consider the differing life situations of older women and men in developing and developed countries. First, there are health differences between women and men, with greater healthy life expectancy among women in both developing and developed countries [32]. Second, there are social differences. Studies in both developed and developing countries have established that social contacts and social support are positively associated with health and wellbeing (e.g., [33–38]) and that older women have higher levels of social contacts and social support than older men (e.g., [1, 33, 39, 40]). However, as women live longer and tend to marry older men, they are more likely to enter widowhood than men and at a younger age [41]. These gender differences in health and social support would be anticipated to influence the extent to which older women and men are present in public spaces.

Furthermore, there are gender differences with regard to media representation. As indicated above, research has shown that older women are particularly underrepresented across a range of media. Feminist critique of media representations suggests that underrepresentation may play a significant role in how women interpret and experience ageing [42]. In addition, in both developing and developed countries, women are generally shown in traditionally feminine and stereotyped roles, and they are often portrayed in a negative and circumscribed manner [43]. For example, one study of Arabic television found that women are more often shown in domestic roles and indoor settings, whereas men are shown in professional roles and outdoors settings (see [44]).

Our primary research question is: how present are older people in public fora in developing countries? A secondary research question is: is the presence of older people in public fora related to their gender? Our site selection, of six cities in six developing countries and one city in a developed country, and the use of two data collection methods were motivated by several considerations. First, as this was an exploratory study it was important to examine the validity, reliability, and feasibility of the methods adopted across a wide range of settings. The selection of a range of developing countries fulfilled this consideration, as such countries would provide challenges and contexts as fieldwork sites that would contrast with those found in the developed country; there would also likely be differences between the selected developing countries themselves. Second, there is a tremendous gap between our understanding of the demographic changes affecting older people in the developing world and our understanding of the public lives of older people in such countries. Our study, with its primary focus on developing countries, would thus both increase the amount of data available on the presence of older women and men in social spaces and in the media in such countries and offer some insight into how their presence in public fora in developing countries contrasts with their presence in similar fora in a developed country, with opportunity for cautious comparison due to the use of standardised methods across fieldwork sites. Finally, the use of three different data sources—non-participant structured observation in social spaces and content analysis of images of older people both in newspapers and on television—would allow for triangulation of data as a way of evaluating the robustness of findings.

## 2. Method

### 2.1. Country Selection

This study involved gathering data in six developing countries—Syria, Jordan, Egypt, Thailand, Kenya, and Tanzania—and in one Western country—the UK—over the course of 2007. In each of these countries, structured observations were carried out in public social places where people were likely to be present and content analysis was performed of images in newspapers and television programmes in which they were likely to be depicted.

Country selection was guided by a need for variation in geographical location, political structures, and ethnic and social mixes, as well as demography. The chosen developing countries offer diversity of cultures, with varying degrees of contrast and comparison with the selected site in the UK. The selected countries represent, respectively, the overwhelmingly Arabic Muslim Syria and Jordan and Hamitic Muslim Egypt; the multiplicity of ethnic groups in Black East Africa, with a predominantly Christian culture and minority Muslim culture in Kenya, and strong Christian and Muslim cultures in Tanzania; and the distinctive, largely Buddhist Thai culture of the Indian subcontinent, with its Chinese and Malay subpopulations. The UK has predominantly a white population with strong Christian heritage but has increasingly diverse ethnic and religious minorities [45, 46]. At the time of data collection, Syria, Egypt, Kenya, and Tanzania had presidential and parliamentary government and Jordan and Thailand had constitutional monarchies with parliamentary government, as did the UK. The urban population is 90 per cent in the UK, 82 per cent in Jordan, 50 per cent in Syria, 43 per cent in Egypt, 33 per cent in Thailand, 23 per cent in Tanzania, and 19 per cent in Kenya [47]. Life expectancy in the selected countries varies considerably. In 2006, it was 79 years in the UK, 72 in Syria and Thailand, 71 in Jordan, and 68 in Egypt, but only 53 in Kenya and 50 in Tanzania. Twenty-two percent of the UK population is over 60, compared to 12 per cent in Thailand, 7 per cent in Egypt, and 5 per cent or less in other countries [48].

### 2.2. Data Collection

The procedures used for structured observation and content analysis are described in detail below. For all data sources, an older person was defined as a person whose appearance suggested an age of 60 or more. For all procedures, weekends in non-Muslim countries and Fridays in Muslim countries were not used for data collection in order to avoid “atypical” days producing biased data.

### 2.3. Structured Observations

A site selection frame was developed for the structured observations of older people. As detailed in Table 1, seven large cities were chosen for observation of social spaces: Sheffield (UK), Haleb (Syria), Amman (Jordan), Luxor (Egypt), Bangkok (Thailand), Nairobi (Kenya), and Arusha (Tanzania). The smallest city observed was Arusha with a population of around 140,000; the largest city was Bangkok with 966,000 [49]. Within each city a central area featuring the main public buildings was selected to ensure that fieldwork sites for observation would be within walking distance of each other. In each city, observations were carried out in seven social spaces: a market, a religious building (e.g., a Christian church/cathedral, Muslim mosque, or Hindu temple, representing the largest denomination in each city), a park, a bus station, a post office, a hotel, and a café. Social spaces were selected to reflect divergence in function, physical layout, open versus enclosed aspect, and expected use by differing subgroups of the population.

A time sampling frame for recording of data from observations was developed based on previous work on the observation of older people in residential care environments [50, 51] and adapted through piloting. For religious buildings, post offices, cafes, and hotels, people going in or out were counted. For markets, parks, and transport stations, people were counted as they passed a particular point including, where appropriate, counting people going on and off buses. Observations were carried out during six phases of the day: 7 am–9 am, 9 am–12 noon, 12 noon–2 pm, 2 pm–5 pm, 5 pm–8 pm, and 8 pm–11 pm. A five-minute period was randomly selected within each phase, during which continuous observation occurred and data was recorded. In each five-minute period, the number and gender of any observed older people were recorded, as were the age and gender constitution of groups involving older people. The five- minute period of data recording balances two conflicting needs: the first is the requirement for a long enough period of behaviour observation to capture the continuous nature of life's ebb and flow and identify the context for activity so as to support the reliable coding of events; the second is the need to restrain the time for observation so as to allow for the accurate recording of often significant amounts of data with accompanying field notes. Breaking up the day into periods also ensured that reliability of the recorded information is not compromised by tiredness, while the overall pattern of life is retained through sampling across an entire day. Information on the validity and reliability of this specific observational data time sampling framework is published elsewhere [50, 51], although it is derived from standard procedures for structured observation methods [4].

Due to local circumstances, some adjustments were made to the site selection frame. Where the city center was non-residential, as in Amman and Bangkok, the old center and the area around the railway station were chosen, respectively, as they are focal points of city life. In Nairobi, the city center was not residential but it was not safe to observe outside this area. In Luxor and Arusha, a main bus stop was selected as the bus station was out of town; in Bangkok the train station was used, as the bus station was in a different area; in Bangkok, Nairobi, and Arusha there were no parks within the area observed so this part of the observation schedule could not be carried out. Some adjustments to the sampling frame were also required to reflect local circumstances. In Nairobi and Arusha, tourists are advised not to go out on foot after dark, so observations could not be done after 8 pm. If a building was not open during a given phase of the day, data could not be collected. Religious buildings, cafes, and post offices were affected by closure. Public spaces selected for observation were in total closed on 11 occasions between 7 and 9 am, on 10 occasions between 8 and 11 pm, on 8 occasions between 5 and 8 pm, and on 1 occasion between 9 and 12 am.

### 2.4. Content Analysis

Media content analysis was undertaken of newspaper pictures and television channels, as detailed in Table 1. Because our study focuses on the visibility of older people, we selected newspapers and television as mass media genres containing a significant component of visual information (as opposed to, say, literature or radio).

#### 2.4.1. Newspapers

A sampling frame was developed in which for each country two local language papers and one English language paper were chosen, unless English was the local language (UK, Kenya, and Tanzania) where two English papers were chosen only. Within this frame, as the researchers had restricted prior knowledge of the countries' media, the newspapers were arbitrarily selected from amongst those papers prominently displayed on at least two news stalls on a given day in each city. Eighteen papers in total were selected. All were dailies and all were published within the relevant country. Circulation and ownership varied.

Within each newspaper, every picture was examined and data extracted on people represented in terms of gender, age group, and number. Where older people were depicted, a brief description of their activity was recorded. The page number and section were noted, together with the headline for English papers and a brief description for other language papers, except when the picture contained only a face with no indication of context. Pictures advertising a product were recorded as adverts and the products were identified where possible. The sections in which the picture appeared were used as base categories for the analysis and finally combined into two superordinate categories: news and non-news.

#### 2.4.2. Television

Within the sampling frame developed for television, terrestrial television channels were prioritized, followed by channels from the region in which the country was situated, and if this was insufficient, a sample of the remaining channels was chosen. Television channels were recorded in all countries but Thailand and Kenya, as in these two countries the researcher was not able to access them within a context that allowed for reliable data recording. In Syria, eight channels were selected, all Arabic. In Jordan, five channels were selected, three of which were Arabic and the other two are Canadian and Turkish. In Egypt, four channels were selected, two Arabic and one American and there was one other channel of unknown origin. In Tanzania, six channels were selected: these were Tanzanian, French African, South African, Indian, British, and American. In the UK, four of the five British terrestrial channels were selected. In total, 27 channels were selected.

The time sampling frame for recording of data from television was developed via adaptation of the frame used for the observation of social spaces described above and via extensive piloting. Each selected channel in a country was studied for a five-minute period during each of the same six phases of the day used for social space observations. A five-minute period was judged, a useful time frame for coding television material as it provides sufficient time to avoid the whole period being taken up by advertisements or credits and to establish reliably the type of programme being broadcast.

During these five-minute periods, every scene was recorded and data extracted on people represented in terms of gender, age group, and number. By “scene,” what is meant is a linear representation or presentation of events or narrative, commencing and ending on an edit or camera switch. Where older people were depicted, a brief description of their activity was recorded. The programme type and where possible the name of the programme were recorded. For programmes in English, the programme type was generally evident through the language. For other channels, the type was decided based on the action and appearance of the scenes. Analytic categories were decided by identifying common programme types in UK and finally combined into two superordinate categories: news or non-news.

As with observations of social spaces, some adjustments to the sampling frame had to be made. It was not possible to observe television from 12 to 2 pm in Jordan, 8 to 11 pm in Egypt, and 7 to 9 am in Tanzania, due to pragmatic constraints on the researcher's access to media during these phases.

### 2.5. Data Analysis

As the data collected was largely non-normal in distribution, nonparametric analytic techniques were used. Parametric analyses were used only where they provided valuable additional descriptive information. Where repeated testing of the same sample occurred, Bonferroni corrections to reduce Type I errors were applied, with an appropriate reduction in alpha for each analysis. Where category cell *n* was too low to enable reliable analysis, combination of categories occurred.

## 3. Results

### 3.1. Social Spaces

In total, 310 older people were observed in social spaces. This was a mean of 1.17 (SD = 4.16, range 0–39) older people per observation period and a median value of 0 (IQR = 0-1) older people per observation period. The number of observed older people per period was therefore greatly skewed toward low values, and older people were observed in only 26.1% of the 264 observation periods. With regard to gender, 184 (59.4%) men and 126 (40.6%) women were observed. In all cities in developing countries, more men than women were observed (developing countries combined: men *n* = 96, women *n* = 7), whereas in Sheffield more women than men were observed (women *n* = 119, men *n* = 88).

The number of older people observed per period varied considerably across cities (see Table 2). Although Sheffield had by far the highest mean number of older people observed per period, this value was inflated by a small number of observation periods with relatively large number of older people. Thus, excluding Arusha (no older people observed), the median number of older people observed per period was highest in Amman (.50, IQR = 0–2.00) and joint lowest in Luxor and Nairobi (0, IQR = 0-0). Due to the very small number of women observed in cities in developing countries and thus the confounding of city status (developed versus developing) with gender, all following statistical comparison between developing country cities and Sheffield was carried out using cases of observed males only.

There was significant variation across cities in the number of older men observed per period (*H*(6) = 38.3, *p* < .001). With regard to the social spaces in which older people were observed, a market was the location in which most older people were observed (*n* = 121), followed by a bus/train station (*n* = 70), a post office (*n* = 67) through to a hotel being the location in which fewest older people were observed (*n* = 4). There was significant variation across social spaces in the number of older men observed per period (*H*(6) = 26.3, *p* < .001); this variation is found in Haleb (*H*(3) = 18.7, *p* < .001) and in Bangkok, Nairobi, Luxor, and Arusha combined (*H*(3) = 14.2, *p* = .003), but not in Sheffield (*H*(3) = 8.80, *p* = .032) or Amman (*H*(3) = 2.30, *p* = .514).

With regard to older people observed per phase of day, the largest number of older people was observed between noon and 2 pm (*n* = 115), followed by 2–5 pm (*n* = 96) and 9 am noon (*n* = 63), with fewer older people observed in the early morning (7–9 am, *n* = 12) and early and late evening (5–8 pm, *n* = 18; 8–11 pm, *n* = 6). There was significant variation across phases of the day in the number of older men observed per period (*H*(5) = 15.4, *p* = .009), but this association was only true for Sheffield (*H*(3) = 17.1, *p* = .001).

There was also considerable variation across cities in the proportion of older people observed who were accompanied as opposed to observed ones alone. While nearly half of the older people observed in Luxor, Nairobi, and Sheffield were accompanied, only 15.8 per cent of older people observed in Bangkok were accompanied, 14.3 per cent in Haleb, and 8.89 per cent in Amman. There was a significant association between the accompanied versus alone status of older men and city (*χ* ^2^(3) = 18.0, *p* < .001), with Sheffield having by far the highest proportion of accompanied older men (42.0%).

Of those observed older people who were accompanied, 84 (79.2%) were accompanied by older people only, while 20 (18.9%) were accompanied by people of other age groups only and two (1.89%) accompanied by mixed age groups of younger and older people. There was a significant association between city status (developed versus developing) and whether or not the accompanying person was old (Sheffield 94.6%, developing cities 40.0%, *φ* = −.66, *p* < .001) with accompanied older people in Sheffield predominantly accompanied by other older people.

### 3.2. Newspapers

A total of 1433 pictures were extracted from newspapers. With regard to the images of people whose ages could be reliably determined, 217 older people were observed in total, in comparison to 513 children or babies, 988 middle-aged people, and 2109 young people. There was thus significant variation in the absolute level of presence of the different age groups (Friedman *χ* ^2^ = 798.2(3), *p* < .001). This variation was significant both for males (Friedman *χ* ^2^ = 336.3(2), *p* < .001) and for females (Friedman *χ* ^2^ = 265.5(2), *p* < .001), excluding children and babies from the analysis (gender not recorded). In terms of the proportion of pictures examined that contained images of people by age group and gender, 88 (6.14%) pictures depicted older men, 27 (1.88%) depicted older women, 938 (65.5%) depicted non-older men, and 385 (26.9%) depicted non-older women. There was thus significant variation in the proportional presence of the different age groups (Cochrane's *Q* = 1616.1(3), *p* < .001). This variation was significant both for males (McNemar's *χ* ^2^ = 747.7(1), *p* < .001) and for females (McNemar's *χ* ^2^ = 317.0(1), *p* < .001).

The proportion of pictures containing older people varied significantly across countries (see Table 3), from 15.3 per cent in Kenya to 2.21 per cent in Jordan (*χ* ^2^(6) = 28.7, *p* < .001). There was also significant variation by country in the proportion of pictures depicting non-old people (*χ* ^2^(6) = 39.6, *p* < .001). For men, there was significant association between country status and depiction in pictures, both for older men (developing country 7.01%, UK 1.71%, *φ* = −.08, *p* = .002) and for non-older men (developing country 67.7%, UK 53.8%, *φ* = −.11, *p* < .001). For women, the association between country status and depiction in pictures was non-significant, both for older women (developing country 1.92%, UK 1.71%, *φ* = −.06, *p* = .830) and for non-older women (developing country 27.3%, UK 24.8%, *φ* = −.02, *p* = .433).

We examined the association between picture content (news or non-news material) and the gender and age of the person depicted. Significant associations were obtained for depiction of an older woman (news 1.51%, non-news 3.43%, *φ* = −.06, *p* = .028) and depiction of a non-old man (news 65.9%, non-news 52.6%, *φ* = .13, *p* < .001). These associations remained significant when controlling for country status.

### 3.3. Television

Content analysis was undertaken of a total of 599 television scenes. With regard to the images of people whose ages could be reliably determined, 106 older people were observed in total, in comparison to 170 children or babies, 370 middle-aged people, and 855 young people. There was thus significant variation in the absolute presence of the different age groups (Friedman *χ* ^2^ = 343.2(3), *p* < .001). This variation was significant both for males (Friedman *χ* ^2^ = 129.8(2), *p* < .001) and for females (Friedman *χ* ^2^ = 177.3(2), *p* < .001), excluding children and babies from the analysis (gender not recorded). Overall, few of the scenes analysed contained older people. Of the 599 analysed scenes, 47 (7.85%) contained older people, while in comparison, 533 scenes (89.0%) contained non-old people. Thirty-four scenes (5.68%) contained older men, while 17 scenes (2.84%) contained older women, and 340 (56.8%) scenes contained non-old men while 252 (42.1%) contained non-old women. There was thus significant variation in the proportional presence of the different age groups (Cochrane's *Q* = 579.3(3), *p* < .001). This variation was significant both for males (McNemar's *χ* ^2^ = 261.3(1), *p* < .001) and for females (McNemar's *χ* ^2^ = 211.4(1), *p* < .001).

The proportion of scenes containing older people varied significantly across countries (see Table 4), from 18.0 per cent in Egypt to 4.24 per cent in Syria (*χ* ^2^(4) = 12.0, *p* = .017). There was also significant variation by country in the proportion of pictures depicting non-old people (*χ* ^2^(4) = 18.3, *p* = .001). Due to the small number of scenes containing older women, developing countries were considered as a whole before examining the association between country status and depiction of people in scenes by age and gender. There were no significant associations between country status and the frequency with which old and non-old people, either males or females, were depicted in scenes.

There was no significant association between presence of older people in scenes and the period of day in which the scenes were broadcast, and there is no significant association between programme type and presence of older people.

## 4. Discussion

Our data, drawn from the structured observations of public social spaces and from the content analysis of images in newspapers and on television channels, is mutually reinforcing: data from all sources indicated a low presence of older people in public fora. Overall, this low presence was particularly true for the developed country sites relative to the developed country site in our study, with women additionally considerably less present than men in developing countries. These findings support the other work that suggests that older people are infrequently visible in social spaces (e.g., [13–15]) and our data echo the claims of a UK Government report that older people are excluded from neighborhoods and leisure activities [35]. There is comparability also with research on media in developed countries that shows older people being underrepresented in advertisements and television (e.g., [18–20, 22, 23, 27, 29]).

One would expect a lower presence of older people in cities in developing countries due to the lower proportions of older people in these countries compared to developed countries. However, when the median number of older people per observation period is considered as the benchmark, Sheffield had a lower median of older people observed in social spaces than Amman. Thus, older people were generally absent from social spaces in both Sheffield and the developing country cities, but only for Sheffield there were a small number of periods when older people were frequently observed. Social spaces were not evenly used by the older people observed, with most older people observed in markets, transport hubs, and post offices. The fact that so few older people were seen in locations more associated with leisure, such as cafes and hotels, is interesting. Research in Western countries indicates that economic and health factors impact older people's leisure activities [52], while being older, being female, and having a limited social network combined with poor finances and poor health reduce participation in activities [53]. While finances may not influence use of parks, poor weather conditions may impact more substantially on the use of open spaces by older, frailer users. It is also interesting that few older people were observed in religious buildings, as churches have been identified as places that are important to older people [13].

Older people in our sample of developing country cities, in comparison to those in Sheffield, are less likely to be accompanied when in public. Leaving aside the very low absolute level of presence of older people in the developing country cities sampled, the finding that older people in these cities are relatively unaccompanied when observed is intriguing. Being unaccompanied may indicate that an older person has a low availability of family relationships, which would place that person at higher risk of poor health, difficulty in accessing social care, and mistreatment and abuse [33–37, 54]. However, being unaccompanied in public may also be interpreted as a sign of social independence and a high level of functioning—no support is sought or needed, rather than being needed and unavailable. Where public social interaction did occur in our study, it was more intergenerational in our sampled developing cities than in Sheffield. This suggests a degree of social segregation by age group in Sheffield. An apparent reliance on age peers for public social activity could place older people at risk of a reduced social network with advancing age. For example, there is a link between spousal bereavement and social exclusion [55]. Older people in Sheffield were significantly more often observed around the middle of the day than in the morning, late afternoon, or evening, but this variation was not present in our sampled developing cities. The relative absence of older people at the beginning and end of the day might be due to older people's fear of the dark and of crime, as identified in previous research in developed countries (e.g., [56, 57]; see also [58], who discuss fear of crime as a domain of social exclusion). Taken overall, the data drawn from observation of social spaces are suggestive of segregation of older people from other age groups (see [59, 60]), a segregation related to place, time, and companionship.

### 4.1. Gender and Presence

When considering older women, 94.4 per cent of those observed were recorded in Sheffield. This confounding of gender with developing versus developed country city status prevented a more nuanced examination of how gender influences women's visibility in social spaces when comparing developed and developing contexts. However, this extremely low level of visibility of older women in our sampled developing country cities does reflect the predominant finding of gender inequalities in such countries (e.g., [61–63]). For example, Lloyd-Sherlock [64] argues that the relative deprivation of older women covers a wide range of aspects, such as entitlements to social policies, increased burdens of care and domestic responsibility, greater likelihood of living alone, and sometimes also discrimination against widows. Research also suggests that women tend to report greater fear of crime than men and that consequences of such fear in terms of the limitation of activities are particularly serious for women and older people [65].

The finding that older men were significantly more likely to be depicted in newspapers from developing countries in comparison to those from the UK, especially when taking population composition into account, suggests less media discrimination against older men in developing countries than in the UK. Previous research on the representation of older people in the media supports our finding that older women were less likely to be present than older men [18–20, 29]. Our finding that younger men were more likely to be depicted in news stories in newspapers and older women more likely to be depicted in non-news items could be seen as a double discrimination of age and gender and points again to older women having very low status within the media, if one accepts the argument that the news underrepresents people of lower status and that this is a gendered imbalance (see also [42, 66]). This finding could also be interpreted in the light of news value theory, in which it is argued that certain factors are associated with events that become news [67]. Most of the “news factors” identified tend to be characteristics of the new event itself rather than characteristics of those individuals involved in the events. However, within news value theory it is argued that an event is more likely to be rendered newsworthy if it involves people belonging to elite groups, who are likely to possess a substantial degree of fame and thereby attract audiences. The low presence of older people, particularly older women, in the media images in our study could thus be indicative of their exclusion from “elite groups” in society, as well as (or alternatively) their exclusion from newsworthy events. Either interpretation connects with an argument that older people and older women in particularly are an excluded social group [68].

### 4.2. Study Strengths and Weaknesses

The most valuable feature of this study is its uniqueness. There are no other directly comparable studies and extremely few of any kind concerned with social integration and exclusion of older people that present data from several countries with differing cultural, political, demographic, and socioeconomical profiles. As this is an exploratory study, it is essential that there is transparency about its methodological successes and limitations so that future research can improve upon this work. Many factors will have influenced the validity and reliability of the data collected and it is important to review these factors.

As mentioned in Section 2, aspects of the sampling frame had to be altered from those originally planned. Such adjustments, made due to local factors in different countries, will have affected the comparability of the data across sites. The choice of fieldwork countries, we have previously argued, reflected a diversity of geographic, ethnic, political, and demographic structures. Pragmatic considerations, such as the resources available to the study, also influenced the selection process, and there is clearly an argument that a different selection of countries (and indeed the selection of different cities within countries) could have produced a different set of findings. Similarly, the selection of social spaces within cities will have produced a unique set of findings—a different set of locations will have presented a different data profile. Nevertheless, the site selection was arrived at following careful deliberation about the meaning of places and their purpose in the lives of individuals and society and represents a sample of locations that are tightly defined both geographically and functionally. As much as any sampling of social spaces drawn from hugely different cultures, we would suggest that there is good reason to think we have attained a reasonable degree of comparability across sites and a robust reflection of the “life” of a city and its peoples. The resources available to the study also required that we emphasize those countries where less research has been carried out—the developing countries. Sheffield, as the single site for data collection in developed countries, should be thought of primarily as a comparator for the data collected from the developing country sites. There is, then, a need for reflection on the extent to which a relatively small number of observations of a few different social spaces, newspapers, and television channels can be interpreted in relation to social integration and exclusion processes on a global scale. Furthermore, while our selection of several different developing countries might have offered an opportunity for disaggregating the effects of ethnicity, religion, and political ideologies on the presence of older people, the small number of older people observed via the three data sources militated against any sophisticated unravelling of such effects, and in many cases in our analysis the data collected from the developing country locations had to be combined.

While acknowledging that carrying out observations in a different set of places within the same cities may well have produced a different data profile, a strength of the study is its capacity to triangulate the data from the three different sources of social spaces, newspaper pictures, and television. The first data source is indeed geographically very tightly defined and therefore highly suspect with regard to any claim for representation of the population from which it is selected. However, the second two data sources are national/regional, not local, and therefore can be argued to be reasonably robust with regard to how they reflect the cultures from which they are drawn. The information that emerges from all three data sources is mutually reinforcing such that the credibility of the data profile drawn from observation of social spaces is strengthened due to its similarity to those drawn from newspapers and television.

Our researcher will have approached the data collection process with conscious and unconscious assumptions and beliefs, which will have been embedded within that researcher's own cultural framework. This cultural anchoring will have affected the data collection process across the different cultures in which it occurred in fundamental and unquantifiable ways. For our study, all data was collected by the same individual. This approach was decided upon partly out of financial necessity and partly to ensure consistency of observations across all sites/countries. The researcher was well prepared through briefing, training, and piloting, and the use of a highly structured observation protocol will have maximized reliability during data collection. However, traditional indices of reliability available when more than one researcher is used during data collection (e.g., interrater reliability) cannot be determined for our study [69]; for further discussion on reliability and validity in observational studies, see [70]. A further limitation arising from our use of a single researcher was that during the observations of social spaces, while the absolute number, gender, and accompanied status of older people were recorded, it was not possible to record the presence of non-old people, or the specific behaviours of the older people observed, as there would have been too many data events to record. Thus, the structured observation method was not used to its full capacity, as has been the case in, for example, research regarding residential care facilities [51].

For all observations, there were difficulties in estimating age. Anyone appearing over the age of 60 was recorded as an older person, and where there was any uncertainty the person was excluded from the “older person” category. Estimates of age were further affected by the distance in observations used to limit the impact of the observer on the observed for social spaces data collection, the researcher's relative unfamiliarity with people from certain ethnic groups, and clothing that obscured faces. Thus, age estimation might be anticipated to be more reliable in the UK than in some of the developing country fieldwork sites. A further issue is whether the age of 60 was a suitable age at which to draw the boundary of old age, given the discrepancy in life expectancy across the sampled countries noted in Section 2. As noted previously, what may at a first glance seem like an absence of older people in developing countries can to some extent be explained by the low proportion of older people in the population, although this does not satisfactorily explain the relative absence of older women compared to older men in public fora. There is an argument that a different boundary for old age should have been set for each country, to reflect variation in life expectancy. Alternatively, a retrospective weighting could have been applied to adjust for life expectancy in the data analysis. We decided that it was preferable to avoid such data transformation and rather present the data as collected but placed within a detailed interpretative context to enable the reader to reach his/her own conclusions.

### 4.3. Conclusions and Implications for Policy

Overall, despite the challenges encountered in this study, it has produced interesting and original findings, which suggest that observational methods and content analysis have utility as ways of exploring social integration and inclusion of older people in both developed and developing countries. While again emphasizing the exploratory nature of our study, there are still broad implications of our findings for policy around older people. Firstly, a lack of presence of older people in public fora implies a lack of representation also in other areas of society such as policy and planning. There is awareness that more engagement is needed with older people in policy and planning, and there have been efforts to achieve this [71, 72]. It should be recognised that a lack of public presence of older people does not preclude a high status for older people in non-public fora or high levels of inclusion in non-public social networks, and this point might be especially pertinent in non-Western and traditional cultures. The role and status of the older person in family networks, for example, cannot be discerned through our data but will vary in nature and significance from culture to culture. Similarly, our study has little insight to offer on the place of older people in rural communities (cf. [73]).

Nevertheless, we would reinforce the notion that a lack of visibility of older people, a public absence of those in later life, has been identified as a form of exclusion [5] and has consequences for how a society configures and represents itself, which in turn will have ramifications for how the role of older people is constructed. Furthermore, this study shows that the exclusion of older people from public fora is not a uniform experience. The emphasis here is that women are more invisible than men, and women in our sample of developing country cities are especially invisible. This suggests exclusion not only by age but also by gender and finally by location, with its variations in demographic, socioeconomic, and cultural factors. If policy needs to address the absence of older people from public spheres, such policy must focus especially on the exclusion of older women.

There may be some concern that moving from a demonstration of the public presence or absence of a social group to the inference that such a group is socially excluded is too great leap. It may be unwise to apply a single model of social exclusion in vastly different cultural contexts without adaption to the local setting (see [74]). Nevertheless, if we accept the United Nation definition of social exclusion as the “involuntary exclusion of individuals and groups from society's political, economic, and societal processes, which prevents their full participation in the society in which they live” [75], then a relative absence from public fora must suggest at the very least a vulnerability to social exclusion, if not actual exclusion itself. We would offer this exploratory study as a starting point for reflection on how best social integration and inclusion can be measured and explored, particularly in locations such as developing countries that are underresearched and which differ in so many respects from the more usual Western sites of social research.

## Figures and Tables

**Table 1:** Observations of older people in public places and in media.

	Data source									
	Public spaces		Newspapers			Television				
	Observations		Read	Pictures examined		Channels watched	Observation periods	Total time	Scenes examined	
	Periods	Time								
	*n*	%	*n*	*n*	%	*n*	*n*	%	*n*	%
City/country										
Haleb/Syria	42	15.9	3	114	7.96	8	6	22.2	165	27.6
Amman/Jordan	42	15.9	3	136	9.49	5	5	18.5	77	12.9
Luxor/Egypt	42	15.9	3	236	16.5	4	5	18.5	50	8.35
Bangkok/Thailand	36	13.6	3	450	31.4	0	0	0	0	0
Nairobi/Kenya	30	11.4	2	170	11.9	0	0	0	0	0
Arusha/Tanzania	30	11.4	2	93	6.49	6	5	18.5	111	18.5
Sheffield/UK	42	15.9	2	234	16.3	4	6	22.2	196	32.7
Total	**264**	**100**	**18**	**1433**	**100**	**27**	**27**	**100**	**599**	**100**

**Table 2:** Observations of older people by gender in seven cities.

	City															
	Haleb		Amman		Luxor		Bangkok		Nairobi		Arusha		Sheffield		Total	
	*n*	%	*n*	%	*n*	%	*n*	%	*n*	%	*n*	%	*n*	%	*n*	%
Men	24	85.7	44	97.8	9	100	17	89.5	2	100	0	0	88	42.5	184	59.4
Women	4	14.3	1	2.22	0	0	2	10.5	0	0	0	0	119	57.5	126	40.6
Total	**28**	**100**	**45**	**100**	**9**	**100**	**19**	**100**	**2**	**100**	**0**	**100**	**207**	**100**	**310**	**100**
Observation period																
Mean	0.64		1.07		0.21		0.53		0.07		0		4.93		1.17	
(SD, range)	(1.45, 0–7)		(1.56, 0–7)		(0.47, 0–2)		(1.06, 0–4)		(0.25, 0-1)		(0.00, 0-0)		(9.32, 0–39)		(4.16, 0–39)	
Median	0		0.50		0		0		0		0		0		0	
(IQR)	(0–0.25)		(0–2.00)		(0-0)		(0–0.75)		(0-0)		(0-0)		(0–5.25)		(0-1.00)	

**Table 3:** Images of people by age and gender in pictures in newspapers from seven cities.

	City							
	Haleb	Amman	Luxor	Bangkok	Nairobi	Arusha	Sheffield	Total
	*n* (%)	*n* (%)	*n* (%)	*n* (%)	*n* (%)	*n* (%)	*n* (%)	*n* (%)
Pictures examined	114 (7.96)	136 (9.49)	236 (16.5)	450 (31.4)	170 (11.9)	93 (6.49)	234 (16.3)	1433 (100)
Picture content								
Older men	9 (7.89)	3 (2.21)	23 (9.75)	30 (6.67)	15 (8.82)	4 (4.30)	4 (1.71)	88 (6.14)
Older women	0 (0.0)	0 (0.0)	2 (0.85)	9 (2.00)	11 (6.47)	1 (1.08)	4 (1.71)	27 (1.88)
Non-older men	69 (60.5)	98 (72.1)	177 (75.0)	293 (65.1)	114 (67.1)	61 (65.6)	126 (53.8)	938 (65.6)
Non-older women	14 (12.3)	27 (19.9)	45 (19.1)	172 (38.2)	49 (28.8)	20 (21.5)	58 (24.8)	385 (26.9)

Note: percentages do not total 100 within countries as some pictures contained images of people from more than one age/gender category while age/gender category could not be determined in other pictures.

**Table 4:** Images of people by age and gender in scenes on television from five cities.

	City					
	Haleb	Amman	Luxor	Arusha	Sheffield	Total
	*n* (%)	*n* (%)	*n* (%)	*n* (%)	n (%)	n (%)
Scenes examined	165 (27.6)	77 (12.9)	50 (8.35)	111 (18.5)	196 (32.7)	599 (100)
Scene content						
Older men	5 (3.03)	7 (9.09)	7 (14.0)	3 (2.70)	12 (6.12)	34 (5.68)
Older women	2 (1.21)	3 (3.90)	3 (6.00)	4 (3.60)	5 (2.55)	17 (2.84)
Non-older men	76 (46.1)	48 (62.3)	26 (52.0)	68 (61.3)	122 (62.2)	340 (56.8)
Non-older women	61 (37.0)	22 (28.6)	22 (44.0)	63 (56.8)	84 (42.9)	252 (42.1)

Note: percentages do not total 100 within countries as some scenes contained images of people from more than one age/gender category while age/gender category could not be determined in other scenes.
